# Treatment of right-sided aortic arch aneurysms with aberrant left subclavian artery with Kommerell’s diverticulum using the frozen elephant trunk technique

**DOI:** 10.1093/icvts/ivad188

**Published:** 2023-11-27

**Authors:** Andrzej Juraszek, Tim Berger, Maximilian Kreibich, Konstantinos Tsagakis, Thanos Sioris, Zeynep Berkarda, Bartosz Rylski, Matthias Siepe, Martin Czerny

**Affiliations:** Department of Cardiovascular Surgery, Medical Center University of Freiburg, Faculty of Medicine, University of Freiburg, Freiburg, Germany; Department of Cardiovascular Surgery, Medical Center University of Freiburg, Faculty of Medicine, University of Freiburg, Freiburg, Germany; Department of Cardiovascular Surgery, Medical Center University of Freiburg, Faculty of Medicine, University of Freiburg, Freiburg, Germany; Department of Cardio-thoracic Surgery, West German Heart and Vascular Center Essen, Essen, Germany; Department of Cardiac Surgery, University Hospital Tampere, Tampere, Finland; Department of Diagnostic and Interventional Radiology, Medical Center University of Freiburg, Faculty of Medicine, University of Freiburg, Freiburg, Germany; Department of Cardiovascular Surgery, Medical Center University of Freiburg, Faculty of Medicine, University of Freiburg, Freiburg, Germany; Department of Cardiovascular Surgery, Medical Center University of Freiburg, Faculty of Medicine, University of Freiburg, Freiburg, Germany; Department of Cardiac Surgery, Inselspital, Bern University Hospital, University of Bern, Bern, Switzerland; Department of Cardiovascular Surgery, Medical Center University of Freiburg, Faculty of Medicine, University of Freiburg, Freiburg, Germany

**Keywords:** Right-sided aortic arch, Kommerell’s diverticulum, Frozen elephant trunk technique

## Abstract

**OBJECTIVES:**

The ideal treatment for aneuryms of aberrant left subclavian arteries with Kommerell's diverticulum arising from right aortic arches remains open.

**METHODS:**

Between January 2015 and December 2020, 5 patients with aneurysms from a right-sided aortic arch with aberrant left subclavian artery and Kommerell’s diverticulum underwent repair by using the frozen elephant trunk technique in 3 aortic centres. Patients’ characteristics were retrospectively reviewed and the surgical procedure and outcomes are presented.

**RESULTS:**

The median age of the 2 male and 3 female patients was 59 (range from 49 to 63) years. The median operative times were as follows: surgery 405 min (range from 335 to 534), cardiopulmonary bypass time 244 min (range from 208 to 280) and aortic clamp time 120 min (from 71 to 184). The mean core temperature was 25.94°C (from 24 to 28). The intensive care unit stay was 4 days (range from 1 to 8) and the in-hospital stay 21 days (from 16 to 34). All patients were discharged and we observed no stroke or spinal cord ischaemia postoperatively. During the median follow-up time of 1003 days (range from 450 to 2306), 3 patients required subsequent thoracic endovascular distal stent graft extension.

**CONCLUSIONS:**

The frozen elephant trunk technique is a good treatment option for patients with aneuryms of an aberrant left subclavian artery with Kommerell's diverticulum arising from right aortic arches. Secondary stent graft extension is a frequently needed component of the treatment concept.

**Figure:**
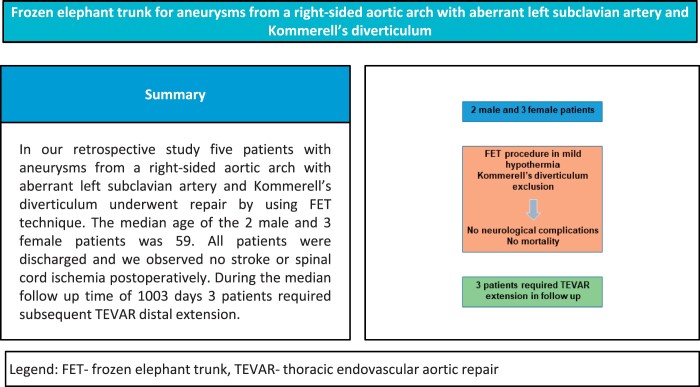

## INTRODUCTION

The most common variant of the aortic arch branching represents ‘the bovine aortic arch’ with the prevalence of 11–27% in the adult population [1]. With a prevalence of 0.4–2.3%, the aberrant right subclavian artery named alternatively arteria lusoria is the most common anomaly of the aortic arch. The aberrant artery usually arises directly from the aortic arch just distal to the left subclavian artery and passes into posterior mediastinum on its way to the right upper extremity [2]. The abnormal vessel course can have important clinical and surgical relevance due to oesophageal or tracheal compression syndromes, important anatomical relations and risk of injury during surgery [2, 3]. The mirror image pathology with an aberrant left subclavian artery arriving from a right-sided aortic arch is far less common [2]. A sac-like widening or bulging of the aortic arch or the descending aorta in the case of an aberrant right subclavian artery is called Kommerell's diverticulum [4, 5].

In case of aneurysmal formation with or without a dissection component, there are several different treatment options including classical open surgical repair, hybrid approaches or even total thoracic endovascular aortic repair (TEVAR) but due to specific procedure-related limitations, none of them emerged as standard treatment strategy. The frozen elephant trunk (FET) technique has become a broader and increasing treatment option for all aortic arch pathologies. This concept allows for both, repair of the underlying aortic disease and rerouting as well as re-vascularization of the supra-aortic branches [6–9].

We report a series of 5 patients with an aberrant left subclavian artery with Kommerell's diverticulum arising from a right-sided aortic arch treated using the FET technique.

## MATHERIALS AND METHODS

### Ethics statement

This research project has been approved by the ethics committee of the University of Freiburg, Freiburg, Germany. Approval number: 20-1302. The reason for formal consent was not obtained due to anonymity of the patients data.

### Patients

**Figure 1:**
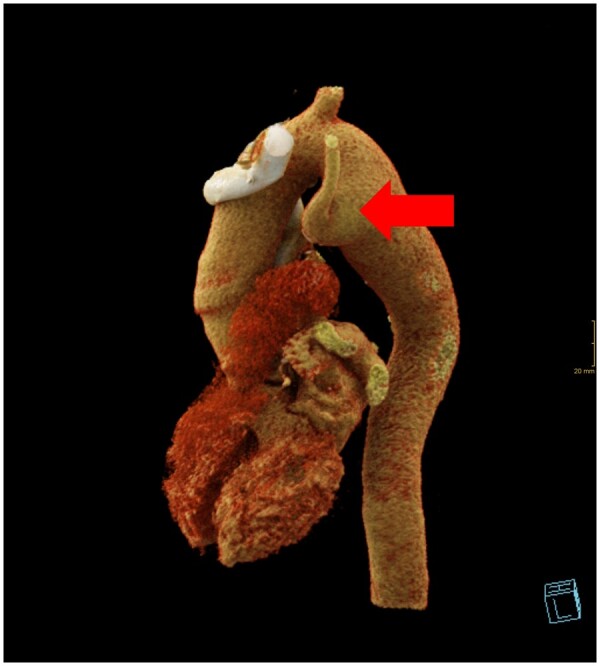
A computer tomography scan reconstruction of aberrant left subclavian artery with Kommerell’s diverticulum marked with a red arrow.

**Figure 2:**
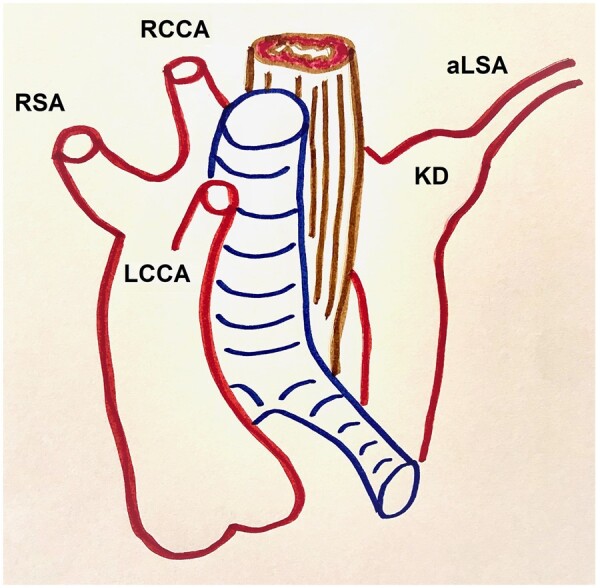
A schematic drawing of aberrant left subclavian artery with Kommerell’s diverticulum.

The prospectively maintained respective aortic databases were screened for patients with right-sided aortic arch aneurysms and an aberrant left subclavian artery originating from a Komerell’s diverticulum on the convex part of the aorta (Figs 1 and 2). Finally, between January 2015 and December 2020, a total of 5 patients underwent total aortic arch replacement using the FET technique in patients with aberrant left subclavian artery. The rationale for the surgery was either tracheal compression of extended vessel size. Two of them were treated at the University Heart Centre Freiburg, Germany, next 2 at the West-German Heart Center Essen, Germany, and one at the Tampere University Hospital Heart Center, Finland. Their baseline characteristics, including previous cardiac surgical procedures, were collected. Their intraoperative data, clinical outcomes after surgery and follow-up data were reviewed. The respective institutional review boards approved this retrospective study, and the need for informed consent was waived.

### Definition of clinical parameters

The primary outcomes were perioperative mortality and stroke. Stroke was defined as a new postoperative physician-diagnosed neurologic deficit persisting >24 hours confirmed by imaging methods—computed tomography or magnetic resonance imaging.

### Surgical technique

**Figure 3:**
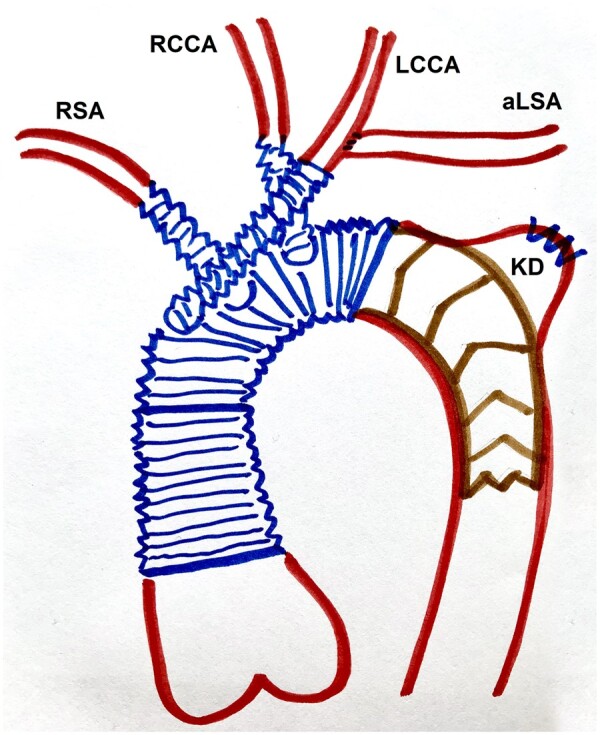
A schematic drawing of the hybrid prosthesis excluded antegrade flow to the Kommerell’s diverticulum.

E-vita open plus (Jotec, Hechingen, Germany) and Thoraflex Hybrid (Terumo Aortic, Inchinnan, UK) prostheses were used. The FET size in dissection cases was the length plus width of true lumen divided by 2 added 10%. In aneurysm cases size in relation to distal landing zone, so that TEVAR extension has a 10% oversize to the distal landing zone with also a 10% oversize to the diameter of the stent graft component of the FET. The surgical techniques from all 3 centres have previously been reported [7, 10, 11]. In our series, we ligated the arteria lusoria distal to the diverticulum to prevent retrograde flow to the aneurysm. The endograft of the hybrid prosthesis excluded antegrade flow to the Kommerell’s diverticulum (Fig. 3). Spinal cord protection with a cerebrospinal fluid drain was used in every case.

### Statistical analysis

Continuous variables are presented as the median and range. Categorical and binary variables are presented as frequencies (*n*) and percentages (%). Statistical analysis was performed using Stata statistical software for macOS (Stata/MP version 13.0; StataCorp, Texas).

## RESULTS

### Baseline and aortic characteristics

The median age of the 2 male and 3 female patients was 59 (range from 49 to 63) years. The pathomorphological substrate was aneurysmal disease in 4 patients and aneurysm on the basis of a residual dissection after previous type A repair in the case of 1 patient. This patient underwent previous supracoronary ascending and hemiarch aortic replacement. The baseline and aortic data are summarized in Table 1.

**Table 1:** Baseline and aortic characteristics

Patient	1	2	3	4	5
Age	49	59	57	63	63
Gender	Male	Female	Female	Male	Male
Aortic pathology	Chronic dissective disease, history of acute type A aortic dissection	Chronic aneurysmal disease	Chronic aneurysmal disease	Chronic aneurysmal disease	Chronic aneurysmal disease
Aortic measurements (mm)					
Aorta ascendens	45	35	33	35	41
Aortic arch	45	32	30	56	31
Aorta descendens segment 1	51	44	37	56	74
Aorta descendens segment 2	37	38	25	34	36
Aorta descendens segment 3	34	27	20	29	34
Previous cardiac surgery	Supracoronary and hemiarch aortic replacement	–	–	–	–

### Operative data

Three E-vita open plus (Jotec, Hechingen, Germany) and 2 Thoraflex Hybrid (Terumo Aortic, Inchinnan, UK) prostheses were used. The median operative times were as follows: surgery 405 min (range from 335 to 534), cardiopulmonary bypass time 244 min (range from 208 to 280), aortic clamp time 120 min (from 71 to 184). The mean core temperature was 25,94°C (from 24 to 28). One patient required re-vascularization of both subclavian arteries performed by aorto-axillary bypasses due to extension of the pathology. Detailed data are shown in Table 2.

**Table 2:** Operative characteristics

Patient	1	2	3	4	5
Cannulation site	Left subclavian	Left subclavian and central aortic	Right subclavian	Right subclavian	Right femoral
Prosthesis	Thoraflex hybrid	Thoraflex hybrid	E-vita open plus	E-vita open plus	E-vita open plus
Distal anastomosis Ishimaru zone	2	2	2	2	0
Additional procedure	–	–	Left carotid-subclavian bypass 14 days prior to surgery	Re-vascularization of both subclavian arteries by aorto-axillary bypass	–
Prosthesis size (mm)	24/26/100	30/36/100	30/130	28/130	36/160
Surgery time (min)	534	360	335	405	420
Cardioplumonary bypass time (min)	260	215	208	244	280
Cross-clamp time (min)	184	120	87	71	156
Core temperature (°C)	24 (rectal)	24,7 (rectal)	28 (bladder)	28 (bladder)	25 (bladder)

#### Detailed surgical procedure (patients 1 and 2)

The left or right subclavian artery was cannulated via an 8 mm side graft. After (re-)sternotomy, the left innominate vein and the supra-aortic vessels were isolated. The target core temperature for hypothermic circulatory arrest was 25°C. Both patients were operated on beating-heart normothermic myocardial perfusion technique. The carotid arteries were clamped and cannulated for bilateral selective antegrade cerebral perfusion. The left subclavian artery was resected at the origin from the Kommerell's diverticulum and mobilized. The diverticulum was closed and the left subclavian artery anastomosed end-to-end to an 8-mm Dacron graft which to maintain the perfusion in the posterior cerebral circulation. The right subclavian artery was also resected and anastomosed end-to-end to an 8-mm graft. A Thoraflex Hybrid graft were deployed and the descending aortic anastomosis was performed in Ishimaru zone 2. Overstenting of the Kommerell's diverticulum offspring was achieved. Afterwards, lower body perfusion was established via the side branch of the arch prosthesis. In the next step, the right subclavian artery was anastomosed to the subclavian branch of the arch prosthesis. After discontinuation of right vertebral perfusion, the artery was anastomosed to the common carotid branch of the prosthesis. The left common carotid artery was anastomosed end-to-end to the innominate branch of the prosthesis and the left subclavian artery was anastomosed end-to-side to the same prosthetic branch.

#### Detailed surgical procedure (patients 3 and 4)

Right subclavian cannulation was used. The aim temperature was 28°C. The left subclavian artery was resected at the origin from the Kommerell's diverticulum and mobilized. E-vita open prosthesis anastomosed to zone 2. Overstenting of the Kommerell's diverticulum offspring was achieved. In patient 4 carotid arteries were re-implanted in the ascending aortic graft using 8-mm polyester grafts. Re-vascularization of both subclavian arteries was performed by aorto-axillary bypass. In addition, the E-vita open stent graft was extended distally by a stent graft in order to exclude the aortic aneurysm over the aortic kinking close to tracheal bifurcation.

#### Detailed surgical procedure (patient 5)

Cardiopulmonary bypass was established via prosthetic 10-mm dacron side branch to the left femoral artery and a central venous cannula after sternotomy. Cooling was targeted to 24°C. Cardioplegia was administered retrogradely using a Custodiol bolus and to the right coronary ostium. Sequence from proximal to distal, of arch vessels takeoff was left common carotid, right common carotid, right subclavian, and left subclavian aortery. First, the left subclavian was exposed at the left border of the trachea, double ligated but not transsected in this case because no dilatation or dysphagia or airway compression was present. An end-to-side bypass was done using a 8-mm dacron prosthesis. The remaining arch vessels were sequentially cut, proximally ligated and individually reconstructed using 8-mm dacron prostheses and perfused with Le Maitres until proximally anastomosed. The ascending aorta was transsected at zone 0. A stiff Jotec (Jotec/CryoLife, Kennesaw, GA, USA) guidewire was advanced to iliac artery level under angioscopic control, through the encoscope working channel. An E-vita open plus 36 mm × 16 cm was inserted, and a Teflon felt reinforced anastomosis made in zone 0. Overstenting of the Kommerell's diverticulum offspring was achieved. A Jotec occlusion balloon was inserted into the endograft, and lower body perfusion begun via the femoral cannula. Rewarming was begun. The ascending aorta was replaced to the sinotubular junction with a 32-mm dacron prosthesis and anastomosed to the trimmed end of the E-vita prosthesis. The sinotubular junction anastomosis was reinforced with Teflon felt. The 4 arch vessel prostheses were anastomosed thus: both subclavian artery prostheses and the left carotid prosthesis were anastomosed to the ascending aortic prosthesis. An arterial cannula was inserted into the ascending aortic prosthesis, de-airing completed, antegrade perfusion commenced and femoral perfusion stopped. The right carotid prosthesis was anastomosed to the side of the right subclavian prosthesis.

#### In-hospital outcome characteristics

The intensive care unit stay was 4 days (range from 1 to 8) and the in-hospital stay 21 days (from 16 to 34). No patient suffered of stroke. One patient developed a left-sided recurrent nerve palsy. The patient cannulated via the femoral artery suffered from a seroma of the femoral cannulation site and delayed wound healing. There was no in-hospital mortality. All patients were discharged home. Detailed data are shown in Table 3.

**Table 3:** In-hospital outcome characteristics

Patient	1	2	3	4	5
Intensive care unit stay in days	6	8	1	4	2
Length of hospitalization in days	20	34	16	28	21
Stroke	–	–	–	–	–
In-hospital mortality	–	–	–	–	–
Postoperative complications	–	–	Recurent nerve palsy	–	Delayed wound healing

#### Follow-up and reinterventions

The follow-up time was 1003 days (range from 450 to 2306). In the follow-up computer tomography scan, no perfusion of the Kommerell's diverticulum was observed. Three patients underwent a subsequent TEVAR extension. In particular, 1 patient had a residual type II and type Ib endoleak. The type II endoleak was treated typically by coiling of an intercostal artery followed by TEVAR extension of the Ib endoleak (Fig. 2). There was no mortality in the follow-up time observed. Detailed data are shown in Table 4.

**Table 4:** Follow-up data

Patient	1	2	3	4	5
Follow-up time in days	653	1003	2280	450	2306
Mortality	–	–	–	–	–
Follow-up events	Uneventful	Uneventful	Minimal type Ib endoleak	Type 1B and II endoleak	Uneventful
TEVAR extension	Elective exclusion of the remaining thoracic aortic aneurysm	–	–	Endoleak treatment	Elective exclusion of the remaining thoracic aortic aneurysm

TEVAR: thoracic endovascular aortic repair.

## DISCUSSION

The FET technique is a good treatment option for patients with aneuryms of an aberrant left subclavian artery with Kommerell's diverticulum arising from right aortic arches. Secondary TEVAR extension is a frequently needed component of the treatment concept.

### Literature background

The main reason for treatment in this group of patients is the presence of symptoms of oesophageal or airway compression. Another reason is the prevention of aneurysm rupture in asymptomatic patients. Limited literature regarding aneurysms from a right-sided aortic arch with aberrant left subclavian artery and Kommerell’s diverticulum is available. It mainly contains case reports. Selected current literature is presented in Table 5 [2, 12–17]. Basically, 3 types of treatment are reported: classical surgical technique by lateral thoracotomy, hybrid treatment using a combination of supra-aortic transpositions and TEVAR, and the use of the FET technique. Kommerell’s diverticulum resection is the standard treatment approach. Vinnakota *et al.* [12] recommended the resection with or without interposition graft of the aorta and left subclavian artery reconstruction for symptomatic adult patients. Despite the high euphoria associated with the use of stent grafts, their limitations and possible complications are now visible. This technique may be associated with endoleak, aorto-oesophageal fistula and retrograde dissection. The right aortic arch in Kommerell’s diverticulum often shows a very steep angulation, and such patients are not appropriate candidates for endovascular repair [2, 12–17].

**Table 5:** Selected literature

Publication authors, yearof publication	Number of patients included	Surgical technique used	Results	Authors’ key comment
Vinnakota *et al.*, 2019 [12]	**152**	87 had no intervention, and 65 underwent open repair (*n* = 55); 19 elephant trunk procedures [9 with aortic dissection], including 7 frozen elephant trunk	There was no operative mortality. Complications included nondisabling stroke (*n* = 5; 8%), tracheostomy (*n* = 3; 4.6%), vocal cord paralysis (*n* = 2; 3%) and reoperation for bleeding (*n* = 3; 4.6%). During follow-up, 3 of 10 patients treated with hybrid or endovascular procedures required reinterventions for endoleaks	Open and endovascular approaches have a high success rate and low mortality risk. Selection of the specific type of intervention should be based on patient anatomy, additional needed procedures and comorbid conditions
Sekine *et al.*, 2015 [13]	9	Replacement of the thoracic descending aorta and *in situ* reconstruction of the aberrant subclavian artery through right (*n* = 6) and left (*n* = 3) thoracotomy	One hospital death due to severe intraoperative cerebral infarction	The standard procedures for Kommerell’s diverticulum are still replacement of the descending thoracic aorta and *in situ* reconstruction of the abberant subclavian artery through a right or left thoracotomy
Gergen *et al.*, 2021 [14]	1	Left carotid-subclavian bypass, thoracic endovascular aortic repair and left video-assisted thoracoscopic surgical division of the aberrant left subclavian artery and vascular ring.	15 months follow-up uneventful	Unique approach addresses left arm re-vascularization, coverage of the Kommerell diverticulum to eliminate risk of degeneration and rupture and thoracoscopic division of the aberrant vessel and vascular ring to alleviate associated compressive symptoms
Matsumori *et al.*, 2020 [15]	1	Single-stage hybrid repair, left carotid–left subclavian artery bypass and embolization of the subclavian artery, followed by replacement of the descending aorta through deep hypothermic circulatory arrest via right thoracotomy	12 months follow-up uneventful	Graft replacement and left subclavian reconstrucition reconstruction is the standard surgical technique. Embolization is safe and effective to minimize backbleeding
Tsukagoshi *et al.*, 2021 [16]	1	Total arch replacement, frozen elephant trunk technique, aberrant left subclavian artery transection and left subclavian artery reconstruction through median sternotomy	Discharged home after 14 days of uneventful hospital stay	Steep transition from the arch to the descending aorta often provides an inappropriate proximal landing zone for stentgrafts. In such cases, the FET technique can help overcome the unfavourable anatomy and concomitantly enables the anatomical reconstruction of the arch branches
Wani *et al.*, 2020 [17]	1	Hybrid endovascular repair. Left carotid/left subclavian bypass, isolation of the left subclavian artery were performed. Isolation of the aberrant left subclavian artery was performed by using a 16-mm Amplatzer, a stent graft was deployed distal to the origin of the right subclavian artery	At 3-month follow-up, the patient’s dysphagia had resolved. Computed tomography scan showed patent stent graft with occluded left subclavian origin and a patent left carotid/left subclavian bypass	In contemporary practice, a hybrid approach with thoracic endovascular aortic repair and plugs is increasingly attempted
Raymond *et al.*, 2019 [2]	1	A left common carotid artery to subclavian artery bypass, thoracic endovascular aortic repair extending across the left subclavian artery origin and placement of an Amplatzer vascular plug distal to the Kommerell diverticulum in the left subclavian artery	Treatment complicated by type IA endoleak, requiring carotid chimney with proximal stent graft extension, and distal aortic dissection, requiring extension of stent graft distal to the level of coeliac artery. Six months follow-up uneventful	An alternative approach to endovascular repair with extra-anatomic bypass as described here is a hybrid endovascular repair using a two-vessel branched stent graft

### The advantages of the FET technique Komerell’s diverticulum treatment

Basically, both stent graft implantation and the FET technique are options available with regard to occluding Kommerell’s diverticulum orifice. However, steep transition from the arch to the descending aorta represents a common anatomy in this patients’ cohort often provides an inappropriate proximal landing zone for stent graft placement. The FET technique providing total arch replacement can overcome the unfavourable anatomy and concomitantly enables sufficient reconstruction of the aortic arch branches [12, 16].

When using FET, 1 aspect is important: how to address with the offspring of the Arteria lusoria, as the Kommerell’s diverticulum offspring is often large. We ligated the arteria lusoria distal to the diverticulum to prevent retrograde flow to the aneurysm. The endograft of the hybrid prosthesis excluded antegrade flow to the Kommerell’s diverticulum. This is the main adventage of the FET in this setting: the hard to be surgically reached Komerell’s diverticulum can be treated by excluding it. Alternatively, a big-sized Amplatzer can be implanted [17].

### Postoperative results

All of the patients in our study were discharged home and we observed no stroke or spinal cord ischaemia postoperatively. In a study of Berger *et al.*, 250 patients underwent total aortic arch replacement via the FET technique between March 2013 and November 2020 for acute and chronic aortic pathologies. In these series, we reported noticeable in-hospital mortality and stroke rate [9]. In fact, the cohorts of these 2 studies are comparable. In the former study patients with all of the pathologies were included. Patients with right-sided aortic arches have no presence of coronary artery disease and arterial atherosclerosis, they reflect the dilatative aortopathy only.

In our another study, 63 patients were treated with FET on for acute or chronic aortic dissection after previous proximal and/or distal open or endovascular thoracic aortic repair. This study reflects a group of patients with dilatative aortopathy with lower incidence of atherosclerosis. The in-hospital mortality was 3%. Postoperative strokes occurred in 8% of patients [8]. Overall, our operating results reflect the experience of FET implantation in previous years and the large volume of our centres.

### Two-staged treatment and reinterventions

During the follow-up time, the descending aortic treatment was completed by TEVAR extension in 3 patients. As previously reported, patients commonly require secondary aortic procedures, emphasizing the need for thorough primary conceptual planning and stringent follow-up. We consider the reinterventions to be the next step in the multi-component treatment of aortic pathology, not the treatment of complications. In our study of the FET technique for the treatment of penetrating aortic ulcers involving the aortic arch in 34 patients, 8 additional endovascular interventions were performed [18].

Basically, the main reasons for secondary interventions after FET implantation are distal stent graft-induced new entry, endoleak and negative aortic remodelling and graft kinking or aorto-oesophageal fistulae. Kandola *et al.* [19] reported the endoleak occurrence after FET of 28% in 25.8 ± 5.7 months of follow-up. 38% of the patients with endoleak needed treatment with TEVAR. The secondary TEVAR should be seen as staged treatment as we do not see distal stent graft-induced new entry in our series.

Singh *et al.* reported on successful intraoperative retrograde stent graft placement to control an endoleak after emergent total arch replacement and FET repair for ruptured Kommerell’s diverticulum and type A aortic heamatoma. The Ia endoleak was managed immediately with proximal extension. Performing of this procedure in a hybrid operating room facilitated optimal management and outcome [20].

### Limitations

The study is limited by the rare occurrence of pathology. The cooperation of the 3 centres allowed to slightly increase the number of reported patients. In our cases, we did not observe tracheal compression after surgery. It cannot be ruled out that the lack of diverticulum resection may not be sufficient in some cases.

To summarize, the FET technique is a good treatment option for patients with aneuryms of an aberrant left subclavian artery with Kommerell's diverticulum arising from right aortic arches. Secondary TEVAR extension is a frequently needed component of the treatment concept.

## Data Availability

Data can be provided upon request. Please contact the corresponding author.

## ABBREVIATIONS

FET: Frozen elephant trunk
TEVAR: Thoracic endovascular aortic repair
